
JOURNAL INFORMATION
==============================
NLM Title Abbreviation: Behav Neurol
Journal ID: Behav Neurol
NLM Journal ID: 8914585
Journal ID: BN

Behavioural Neurology

ISSN: 0953-4180
EISSN: 1875-8584
Publisher: Wiley

ARTICLE INFORMATION
==============================
PMCID: PMC5434363
PMID: 20714056
DOI: 10.3233/BEN-2010-0272
Article ID: 495061
Article version: 1
Subjects: Editorial

Trends in Cerebellar Research

Desmond John E. *
Division of Cognitive Neuroscience Johns Hopkins Medical Institutions Baltimore MD USA
*John E. Desmond: dr.jdesmond@gmail.com
Print publication date: 2010
Electronic publication date: 2010 Aug 16
Volume: 23
Issue: 1-2
First page: 1
Last page: 2
Received 2010 Aug 12; Accepted 2010 Aug 12
Copyright: Copyright © 2010 Hindawi Publishing Corporation and the authors.
Copyright year: 2010
License: This is an open access article distributed under the Creative Commons Attribution License, which permits unrestricted use, distribution, and reproduction in any medium, provided the original work is properly cited.
License URL: https://creativecommons.org/licenses/by/3.0/

==============================
Behavioural Neurology 23 (2010) 1–2
DOI 10.3233/BEN-2010-0272
IOS Press

Editorial

### Trends in Cerebellar Research

John E. Desmond*
*Division of Cognitive Neuroscience, Johns Hopkins Medical Institutions, Baltimore, MD, USA*

This issue of Behavioural Neurology is devoted to the investigation of cognitive contributions of the cerebellum. The fact that such a special issue is appearing in this journal, with similar issues also appearing in at least two other journals this year, reflects a salient trend in cerebellar research that began in the mid 1990's. This trend, towards an increasing focus on the cerebellum's role in cognition, is evidenced by an analysis of articles obtained in the Pubmed database pertaining to cerebellar research over the past four and a half decades.

First, to identify research that had a focus, at least in part, on the cerebellum, articles containing *cerebell*, cerebrocerebell*, or cerebro-cerebell* in the title were identified (where * denotes a wildcard character). These articles were divided into human and animal studies using the Pubmed "limit" feature. For both the human and animal articles, studies were classified in one of three categories. The first was a *cognitive* category, containing one or more of the following words in the title: spatial, mental, emotion*, affective, reasoning, language, linguistic, planning, fluency, cognit*, memory, attention*, executive, non-motor, or neuropsych*. These terms were designed to identify articles pertaining to higher-order cognitive function. The second was a *motor/cognitive* category and these articles had none of the cognitive category title words but did have one or more of the following words in the title: timing, learning, conditioning, or speech. These terms were designed to identify articles that may reflect motor functions of the cerebellum, but also have implications for cognition. The third was a purely *motor* category, and these articles had in their titles none of the cognitive or motor/cognitive words but did contain one or more of the following words: motor, sensorimotor, oculomotor, movement*, postur*, balance, gait, gaze, saccad*, nystag*, locomot*, or walking. Articles were manually inspected after the search to remove inappropriate articles. For example, the title word "spatial" had a tendency to find articles pertaining to the spatial distribution of anatomical projections to the cerebellum from other brain regions, as well as articles pertaining to spatial orientation or spatial memory functions of the cerebellum. These purely anatomical studies of the cerebellum were removed from the search results.

It can be seen in Fig. 1 that prior to 1990, for both human and animal studies, articles concerning purely motor functions of the cerebellum completely dominated the cerebellar literature. Around 1985 the involvement of the cerebellum in classical eyeblink conditioning was discovered, and 42% of the motor/cognitive animal articles after that date pertain to classical conditioning. In the human literature the growth in interest in motor/cognitive cerebellar research has been more gradual, but the proportion of such articles has risen considerably in the last 10 years. It is also noteworthy that the number of investigations of motor functions of the cerebellum is also showing an increasing trend in the human literature, while the numbers have remained mostly stable in the animal literature for decades.

For research articles pertaining to human cerebellar cognitive function, Fig. 1 shows that prior to 1990 a relatively small proportion of articles were devoted to this topic. However, a sharp increase in interest

*Address for correspondence: John E. Desmond, Ph.D., Division of Cognitive Neuroscience, Johns Hopkins Medical Institutions, 1620 McElderry Street / Reed Hall East – 2, Baltimore, MD 21205, USA. Tel.: +1 410 502 3583; Fax: +1 410 502 2189; E-mail: dr.jdesmond@gmail.com.

ISSN 0953-4180/10/$27.50 © 2010 – IOS Press and the authors. All rights reserved

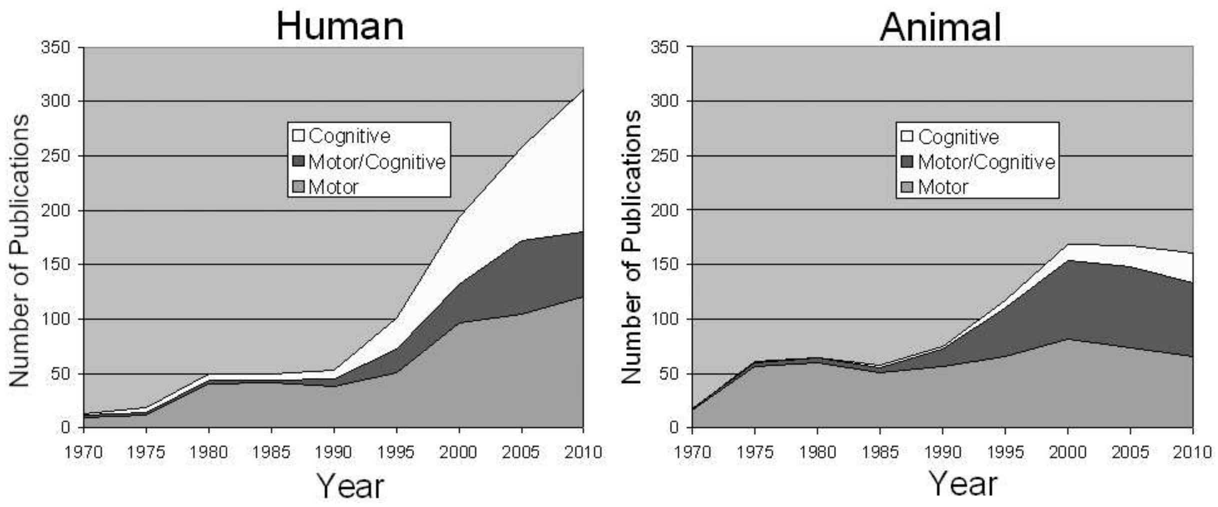

Fig. 1. Trends over time in human and animal publications pertaining to cerebellar function. Articles were classified as to whether they investigated cognitive, motor/cognitive, or motor functions of the cerebellum, using article title search criteria indicated in the text. The number of publications depicted at each year point on the X axis represents the counts of articles in the 5 years prior to that date. So for example, on the Animal graph, the number of publications appearing above the year 2000 is 168. This represents the number of animal articles whose year of publication was less than 2000 and greater than or equal to 1995. A total of 81 motor articles, 73 motor/cognitive articles, and 14 cognitive articles were obtained during that 5-year span.

began between 1990 and 1995. The timing of this increased interest corresponds with the initial reports of the feasibility of functional MRI for exploring human brain function in 1991. Interestingly, only about 10% of the post-1991 human cognitive cerebellar articles represented in Fig. 1 directly involve functional MRI methodology. However, the true impact of the widespread availability of functional MRI on cerebellar research is more difficult to quantify. That is, Fig. 1 only reflects articles that have a main focus on the cerebellum (as indicated in the title) and does not represent the numerous functional MRI papers that were oriented toward whole-brain investigations and were focused on cognitive processes rather than specific anatomical structures. Among these cases, there are a considerable number of studies in which cerebellar activation was found during high-level cognitive tasks such as random number generation, dual task performance, Wisconsin Card Sorting, syllogistic reasoning, and theory of mind operations. These reports no doubt provided motivation for numerous clinical and patient researchers to investigate to what extent these neuroimaging-derived cerebellar activations are relevant to a patient's mental status. The net result is that in the past 5 years, despite the rising trend in human cerebellar motor research, and for the first time ever, the number of human articles pertaining to cerebellar cognitive function exceeded the number of articles devoted to purely motor function.

In addition to the influence of functional neuroimaging and the ubiquitous presence of cerebellar activation in cognitive tasks, the explosive growth of interest in cognitive functions of the cerebellum in the last 15 years likely reflects two additional factors. The first is that, as pointed out by Timmann and Daum in the present volume, cognitive effects of cerebellar damage can be somewhat variable. Identifying the sources of this variability will likely provide important clues as to the role of the cerebellum in cognition, but in the meantime, replication and confirmation of a variable phenomenon is an appropriate and important safeguard for the scientific process. The second is that many researchers may be transitioning from asking *if* the cerebellum has a role in cognition to asking *how* it performs that function. The growth curve of Fig. 1 may therefore reflect in part a phase of scientific "conjecture and refutation" to see if there is one parsimonious and satisfying explanation that ties together all of the evidence of cerebellar involvement in both cognitive and motor function. Keeping in mind that parsimony is a preference rather than a law of nature, the growth depicted in Fig. 1 nevertheless suggests that the next 5–10 years should prove to be interesting for cerebellar research.
